# Supplementary material for: Access to healthcare for people with disabilities in Zambia: a qualitative study
Source: PLOS Glob Public Health. 2025 Mar 31;5(3):e0004145. doi: 10.1371/journal.pgph.0004145 (PMC11957284; doi:10.1371/journal.pgph.0004145)
Supplement: S1 Table — (DOCX) [file pgph.0004145.s001.docx]

**S1 Table: Overview of key themes and additional quotes**

| **Levesque component** | **Theme** | **Quotation** |
| --- | --- | --- |
| Approachability, Ability to perceive | Improving information on healthcare | *“Let’s say those who may not notice their children, I think maybe if they come together if we had... if we run such programmes, I think it would be helpful in child development. And let’s say they come together, meet, they're being taught on how to manage their children; I think that would be helpful.”* (Clinician, Chongwe) |
|  | Perception of support available | *“When the doctor came, he spoke to my sister and immediately said, and these words did not please me (begins to cry), but he said, “this is not a disability hospital!”. I could hear everything he was saying and I was very hurt so, I thought to myself that us disabled people, are we supposed to have our own hospital and not mix with other people or what? It really hurt me a lot”* (Adult with a physical impairment, Chongwe)  *“In our local clinic, sometimes we would take the child there, then they will just say, ‘No, since the child has got cerebral palsy we can't attend to her, take her where you usually take her… we can't attend to her.’ […] I would prefer to go to a local clinic because it’s near. Now because of their attitude they... they've nothing to do with children with cerebral palsy.”*  (Caregiver a child with a physical and intellectual impairment, Lusaka) |
|  | Limited outreach targeting people with disabilities | *“…persons with disabilities are not accessing [healthcare], and because they [healthcare services] don't see persons with disabilities at these facilities, they assume that they are not there.”* (Key informant, disability organisation) |
| Acceptability, Ability to seek | Negative attitudes towards people with disabilities | *“The first thing they'll see is the disability on the child, so some of them will just sit there and it’s like they're watching a movie. So now I have to be bold enough to speak, but sometimes it’s so frustrating when you go that side and then people are just looking at you like, ‘what has happened?’. They look at you... they're looking and then they're looking at the child, ‘what happened, what happened?*’ (Caregiver of a child with a physical and intellectual disability, Lusaka) |
|  | Professional values and knowledge: traditional healers | *“…they tell people lies. They deceive a lot of people. Because when you go to them, instead of just telling you to take the child to the hospital they will see it as an opportunity to just make money. They will tell you a number of lies, like the child was bewitched by that neighbour of yours who you had a feud with. Such lies. So they really need such information for them to know that such children with disabilities exist, it is God alone who created, yes.”* (Caregiver of a child with a physical and intellectual disability, Chongwe) |
| Availability and accommodation, Ability to reach | Challenges with transport and inaccessible environment | *“I do manage but I face challenges, especially that of transport when I walk with him on my back. Now he has grown and is heavier, I have to take stops on the way just to catch a breath.”* (Caregiver of a child with a physical impairment, Kafue)  *“Then the (name of hospital), you're talking about the transport to the other side [of Lusaka] […] so if you're talking about transport you're talking about also the cost, so sometimes we just have to stay home and do one or two things.”* (Caregiver of a child with physical and intellectual impairment, Lusaka) |
|  | Inaccessible facilities and environment | *“It is usually… very difficult [to take her to the health centre]. This is due to the fact that (Name of child) is grown, therefore having to carry her on my back is challenging. Despite having this wheelchair that we were given by (Name of hospital), it is difficult to use it because of the state of our road. It is a sandy road… it is difficult to push a wheelchair in the sand road. So the alternative is to carry her on my back.”* (Caregiver of a child with a physical and intellectual disability, Chongwe) |
|  | Communication difficulties and inaccessible health information | *“Nurses and doctors use writing as a way of communication… but I haven’t… I am not a learned person and I don’t understand […] So now even writing does not help me to communicate… so the nurses they try to write, but because I haven’t been to school I don’t understand what they write. So it’s better for them to proficient in sign language and there is sign language interpreter […] All government and private institutions, they must engage sign language interpreters so that the message can be clearer, between I and also the health provider.”* (Deaf adult, Kafue) |
| Affordability, Ability to pay | Unaffordable direct costs | *“Participant: At the hospital, the help wasn’t much, because when we go, there is no medicine. So they just prescribe and tell you to buy and mostly we don’t have money. I have been there I don’t know how many times and they never gave me the drugs. Instead, they just prescribe, so it becomes a challenge. When we go there, we are attended to just fine but the issue of drugs… there are usually no drugs. They usually just give panadol and say we should buy the rest. The drugs that seem to be helpful are the ones we are told to buy.*  *Interviewer: Were you able to buy (the medication)?*  *Participant: No I didn’t buy. I cannot lie.”*  (Caregiver of a child with hearing impairment, Kafue) |
| Appropriateness, Ability to engage | Lack of training on disability for health providers | *“I think maybe in our training you should even include the component of how to manage these people in our communities, like basic training in physiotherapy. Yeah, because if we can have that, it will help us a lot. When you train us, then you train the community. They are... those people are gatekeepers, you train them, and the volunteers will be trained, our community will move from point A to go to another level dealing with their disabled people […] So if also we are trained in how to do basic… simple physiotherapy, we will help the community, because once we are trained, we will make sure we streamline to the community, yes. Once the community has the knowledge, I know, people with disabilities will benefit a lot.”* (Nurse, Chongwe) |
