# Supplementary material for: Access to healthcare for people with disabilities in Zambia: a qualitative study
Source: PLOS Glob Public Health. 2025 Mar 31;5(3):e0004145. doi: 10.1371/journal.pgph.0004145 (PMC11957284; doi:10.1371/journal.pgph.0004145)
Supplement: Attachment — Submitted filename: Response to reviewers.docx [file pgph.0004145.s004.docx]

**Reviewer #1:**

I really enjoyed reading this article. Highlighting the perspectives of individuals with disabilities is crucial, as their voices are often marginalized even in high-income countries (HICs) with greater resources. Providing a platform for these voices adds significant value to both the research and the broader conversation on inclusion and accessibility.
Regarding the feedback format, I’ll offer a general comment on each section, followed by specific suggestions aimed at enhancing the quality and clarity of the work.

**Thank you for your feedback. We greatly appreciate the time you have put in for a thoughtful review. Please see our response and actions listed below. Please note that the line numbers refer to the version with tracked changes and ‘All markup’.**

Abstract
• The abstract is well-structured and provides a comprehensive overview of the study. I recommend removing the last sentence in the background section, ‘This study employs qualitive method to generate new evidence’, as this content belongs more appropriately in the methods section. Instead, this part could be rephrased as: ‘In this qualitative study, we conducted in-depth interviews with 48 participants…”.

**Amendment made as suggested (line 29 and 31).**

Introduction
• The introduction provides valuable context on the topic and is well-proportioned in length. However, the phase ‘than people with disabilities’ appears repeatedly throughout the section and may lead to redundancy. For better fluency, I suggest removing this phrase on line e58 when discussing all-cause mortality.

**We have removed the reference as suggested (line 75).**

• Additionally, the introduction could benefit from a smoother transition between the second and third paragraphs. Adding 'especially in LMICs' to the end of the sentence concluding with 'and improve health outcomes for people with disabilities' on line 63 may strengthen this connection.

**We have included your suggestion above (line 80). To add further context, we have added that the majority of people with disabilities live in low- and middle-income countries (line 64).**

• Currently, the connection is made with the study’s objective, but it might be more effective to place the objective at the end of the introduction where it naturally aligns with lines 88 and 89. I suggest revising the objective to clearly encompass the study’s focus on barriers and facilitators to access, as well as potential solutions, as discussed in the results.

**We have deleted reference to the objective (line 80). We have added additional detail to the study aim, highlighting the focus on barriers, facilitators and solutions (line 107).**

Method
• The methodology is well-explained and, while qualitative research may not follow strict standardization, it provides sufficient detail for replicability.

Study setting
• The classification of location is somewhat overlapping here, as it differentiates people by both district within Lusaka province and location type. From what I understand, the three districts were chosen to represent urban, peri-urban, and rural locations; however, only Lusaka is entirely urban, while Chongwe and Kafue are mixed. To improve clarity, I suggest acknowledging the participants' districts of origin but using location type as the sole classification and removing the district category from the table.

**We have removed district from the table.**

• Additionally, consider standardizing terminology throughout the text, using either 'location' or 'locality' consistently.

**We have amended terminology to be consistently ‘locality’.**

The section also includes a description of Zambia’s healthcare system structure. While relevant, this information may fit better in the introduction, especially since all health professional participants are from primary healthcare settings. Summarizing it briefly in the introduction will better set the context.

**We believe this is useful information and believe it is appropriate here, under study setting. We are concerned that this information in the introduction will detract from the focus on rationale. We request to leave it as is.**

Participants
• It may be helpful to explain the rationale behind the classification of disability used in the study. For instance, why was there a differentiation between visual and hearing disabilities, rather than grouping these as sensory disabilities? A brief explanation could help readers understand this choice.

**Additional information added to clarify the classification (line 145).**

Data collection
• For clarity, please ensure a logical order of information. For example, details on the language used in interviews (lines 144–148) would be more coherent in the third paragraph (around line 160), as it relates more closely to procedural specifics.

**Amendment made as suggested in order to create a more logical flow. Information on language of interviews now provided at line 190.**

There appears to be an important limitation concerning adult participants with intellectual disabilities. Using caregivers as proxies may not provide accurate insights; literature indicates that caregivers' reports on health experiences do not always align with the actual experiences of individuals themselves. For an example of this topic, please see [https://jamanetwork.com/journals/jamapediatrics/fullarticle/2769779](https://url.uk.m.mimecastprotect.com/s/7hRICl714TyzPolcgFkIzH2do?domain=jamanetwork.com)
When direct communication with participants was not possible, this represents a barrier that should be recognized as a limitation. If participants' intellectual disabilities prevented complex communication even with augmentative speech strategies, the role of caregivers as informants might be acknowledged as a separate participant category, such as 'Caregivers of Adults with Disabilities.' Including this as a limitation could strengthen the study's transparency.

**We have included additional information in the methods (line 202). We have also added as a limitation (line 763).**

Data analysis
• The decision to use the Levesque Framework for healthcare access is well-suited to the study's objectives. Along with the figure, consider mentioning the framework's main components and key themes within each part to give readers a clearer understanding of its application in this context.

**We have added this information throughout the results.**

Reflexivity
• I appreciate the inclusion of this section, as it shows the authors’ commitment to addressing potential biases. It may be more appropriately placed immediately after 'Ethical Considerations,' and I suggest revising the title to more precisely reflect the content.

**We have moved this to the end of the methods section, as suggested. We have clarified that this is reflexivity of the research team (line 254).**

Results
• The results presented in this paper are highly engaging, although I have two main concerns. First, the term 'participant' is used ambiguously throughout. Given the various participant categories, it would be helpful to clarify each instance to indicate whether it refers to all participants or specific categories. Authors have done it in most cases but others remain ambiguous.

**We have added clarification that participants refer to all participant groups (line 274). We have aimed to be specific when talking about a single group.**

I also suggest refining the language of the results section to clearly convey that this information reflects the study’s findings. Using verbs such as 'reported,' 'noted,' 'mentioned,' or 'stated' may enhance readability and provide consistency in reporting participants' perspectives. Frequently, it is confusing whether the statement comes from the results or it may be an assumption.
**We have included a statement to clarify that the findings come from the study participants, rather than presentation of information from the wider literature or author assumptions (line 274).**

To improve clarity for readers, consider renaming and numbering the themes, e.g., 'Theme 1: Ability to Perceive and Approachability.' Additionally, briefly specify Levesque’s categorization within each theme (e.g., supply-side vs. demand-side). Within each theme, short subtitles could be added at the start of paragraphs, formatted in italics, to guide readers through key points. Here’s an example reformulation for theme 1 following this structure:
“Theme 1: Ability to Perceive and approachability”
General health literacy. People with disabilities and caregivers discussed two types of healthcare – general primary healthcare (e.g. under-five, vaccines) and disability-specific services [deleted redundant info]. [Deleted ‘while’] Participants reported general health literacy and many lacked information and knowledge on disability, disability-specific services and referral system, especially in rural areas. They [participants and caregivers] also noted that primary healthcare services do not provide disability support and they do not attend these facilities when they have a concern related to disability [deleted information about referrals, noted before], leading to underutilization of available services.
Additionally, people with disabilities reported being refused support at primary health services for general health issues and, accordingly, many primary health professionals reported seeing their role for people with disabilities as referral to secondary and tertiary care only. [Deleted information about transport because it belongs to another theme].
(quote)
Systems outreach. Health professionals….”
**We have amended the headings to include ‘Theme 1’ etc. We have also added in sub-headings as suggested throughout the results.**

Ability to Perceive, approachability
• The information beginning on line 253 with 'For example' seems more relevant to the discussion section. For instance, the phrase 'some organizations have aimed to work with primary health facilities' does not appear to be directly derived from participants' responses. Clarifying whether this information is part of the study’s findings or general context would be helpful.

**This information came directly from participants. We hope the statement at the start of the results provides clarification.**

• Similarly, the third paragraph of this theme would benefit from a focus on participants’ suggestions regarding solutions to the two barriers highlighted earlier in this theme. Additional context could be reserved for the discussion.

**We confirm that all information on suggested solutions provided here is from participants.**

Ability to Seek, acceptability
• On line 277, it would be helpful to clarify if participants directly reported the negative impact discussed.

**This was directly reported by participants. New statement at start of results aims to make this clear.**

• The statement 'Participants recommended that headmen and volunteers be trained to improve disability awareness in communities' appears to overlap with content in the previous theme and may not be needed here.

**Slightly different solutions presented, as the first reference is with regards to health information and health access, predominantly. The second is about more general disability sensitisation among the wider community. We have therefore not made any amendment.**

• Consider including a quote after the first paragraph of the theme to strengthen the participants' perspectives. Then, instead of explaining the quote (lines 292–294), a summary, such as 'addressing the patient directly rather than talking to an assistant,' might add clarity.

**We have removed the explanation of the quote (line 372). We have not included a new quote, as there are already two quotes under this theme highlighting negative attitudes and limited knowledge from the patient perspective.**

• Paragraph 7 (line 304) and its associated quote repeat information from the previous theme on training, which may lead to redundancy.

**We reviewed this quote and believe it is better aligned to outreach. We have moved it to line 318.**

Ability to Reach, Availability and Accommodation
• The second paragraph could be restructured for clarity, focusing on the information flow. Here’s a suggested revision: 'Participants regularly cited transportation as a significant barrier due to both availability and cost, particularly in rural and peri-urban areas where primary health facilities are often distant from people’s homes. In these regions, challenging terrain, such as rocky or sandy roads, made travel difficult for those with physical or visual impairments. Even in urban settings, narrow and uneven pavements limited accessibility for people with disabilities.

**Thank you for the suggestion. The amendments have been made as suggested (line 441 and line 451).**

• After this paragraph, consider grouping insights by topic: first, discuss transportation costs, then accessibility challenges, and finally, explore solutions, including those appropriately reported as loss of dignity or challenging (e.g. asking for money).

**We believe that the new sub-headings have now made this section more clearly structured.**

• In paragraph 8 (line 390), results are presented in the present tense rather than the past, which may disrupt consistency. Also, rather than a detailed example, consider a summarized version to contextualize the quote more effectively.

**Amendments made (line 506).**

• In paragraph 11 (line 422), 'Participants called for Braille on medication packets and bottles,' would read more clearly with the addition of 'and suggested other simple solutions, such as…

**Amendment made (line 534).**

• On line 434, confirm if ‘This increased risk and stress’ is participant-reported.

**Yes, this comes from participants. The statement at the start of the results hopefully clarifies this.**

• Lines 439-440 contain repetitive language: ‘There were calls’ appears twice and could be changed for fluency.

**Amendment made (line 553).**

• To avoid redundancy, consider removing detailed descriptions of disability assessment procedures (lines 444-448).

**We believe that this information is an important finding, as it limits accessibility of the disability card, and we have therefore not removed it.**

• In the final paragraph of this theme, ‘people’ is mentioned but it’s unclear who this refers to —clarifying this term would improve understanding.

**We have moved this paragraph to line 593 and amended to participants.**

Ability to pay, affordability
• In the second paragraph, delete 'In addition to issues with transport cost, discussed above,' as it’s close enough to refer back without additional prompting.

**Deleted as suggested.**

• Subheadings within this theme, such as 'Social Support,' 'Medication Affordability,' and 'Service Affordability,' may also help guide the reader through key points.

**We have added new headings as suggested.**

Ability to engage, appropriateness
• On line 484, consider whether the 'medical or social view of disability' is a reported outcome from participants or a conclusion drawn from results.

**We have made this clearer (line 613).**

• At the end of page 20 (line 492), add an 's' to the end of 'condition' to maintain grammatical accuracy.

**Amendment made (line 627).**

• In the third paragraph of this theme, the example of training by NGOs would be more suitable in the discussion.

**This information came from participants and is suitable to leave in the results.**

• In the subsequent paragraph, clarify if the information about the government is reported by participants or drawn from literature (if the latter, it should be included in the discussion instead).

**We confirm that this information is from participants and trust that the statement at the start of the results provides clarification.**

Discussion
• The discussion could start with a concise summary of findings that directly addresses the study’s objectives. Consider restructuring to better highlight where this study’s findings align or diverge from previous literature. Currently, literature alignment is acknowledged but not explained, so expanding on this could enrich the discussion. Additionally, incorporating the points identified for discussion during the results presentation would provide valuable context here.

**We have provided more detail on findings consistent with the wider literature, especially in the recent meta-synthesis which also utilised the Levesque Framework (line 687).**

Strengths and limitations
• This section is well-articulated, with clearly acknowledged limitations. I recommend including the limitation regarding caregiver proxies as noted earlier in this review.

**We have made this addition, as suggested (line 763).**

Additionally, the final statement would benefit from a more nuanced reflection on gender roles: although women are recognized as the primary caregivers, this largely reflects a broader, patriarchal social structure rather than a unique factor within this context.

**Although this is a valid point, we do not feel we can do this discussion justice in this paper and have not included additional information on this point.**

• The observed underrepresentation of male caregivers may be linked to limited paternal involvement in child-rearing, which has implications for children with disabilities. Research in high-income countries (HICs) suggests that when fathers are less involved, it can negatively affect early child development—a critical stage for children with disabilities, where many intervention programs are focused.

**We have added this information (line 761).**

• In terms of access, this gender perspective reveals how barriers faced by women in accessing healthcare may indirectly restrict children’s access as well. Women, who often advocate for their children in healthcare settings, may find their voices overlooked or mistreated, potentially hindering timely and appropriate care for children with disabilities.

**Additional information provided (line 704).**

**Reviewer #2:**

The authors conducted a qualitative research study in which they performed in-depth interviews about healthcare access for people with disability with 48 participants, including 16 adults with disabilities, 16 caregivers of children with disabilities, 12 primary healthcare professionals, and four key informants from government and civil society. Participants were recruited from three districts in Lusaka Province: Lusaka, Chongwe, and Kafue. Key themes were mapped against the Levesque framework of healthcare access, and the authors presented various key findings regarding the difficulties faced by this population in that specific area of Zambia.

This work presents novel and valuable findings and offers insights into how institutions in Zambia can effectively approach and enhance healthcare systems to improve accessibility, approachability, and acceptability, among other factors. It is well written and contributes significantly to the field. I commend the authors for their demonstration of cultural humility in the reflexivity section of the paper, where they emphasize how a diverse group of authors can contribute to the advancement of the academic field, and I was also impressed by their acknowledgment of the challenges in their study, particularly regarding the lead author's background, along with their efforts to address these issues. Below are a few comments listed.
**Thank you for your thoughtful feedback. We very much appreciate the time taken. Please see our response and actions listed below. Please note that the line numbers refer to the version with tracked changes and ‘All markup’.**

Comments

• The sample characteristics in Table 1 could include the primary language spoken or the language in which the interviews were conducted. It would also be beneficial to add the ages of caregivers, healthcare professionals, and key informants.

**We have made the suggested amendments to the table.**

• Could you expand on the process of developing the interview guides and indicate whether there were differences among the guides used for different groups or languages?

**Additional information added (line 187).**

• It is stated that the interviews were conducted in English, Cinyanja, or Icibemba. Could you explain and provide data on how many interviews were conducted in each language and describe the process for determining when interviews would be conducted in English?

**We have provided additional information in the methods (line 190) and table 1. We incorrectly included Icibemba at first submission and have amended.**

• In the methods section, it is mentioned that “ …authors developed a coding framework, with transcripts coded in NVivo 12 by NS.” Could you clarify whether NS was the only coder? Additionally, could you specify whether an intercoder reliability process was followed, and if so, provide information regarding this?

**We have included additional information (line 219). We have included a limitation that the coding was not completed by two coders (line 753).**

• The methods section states that “two adults with disabilities were interviewed with a caregiver providing a proxy response.” Could you please explain the interview process involving proxy responses in more detail?

**We have made it clear that by proxy we mean a caregiver interviewed alone providing proxy response on their relation’s experience (line 201).**

• It would be beneficial to present the sex of the respondents along with the quotes.

**We have added this information throughout the results.**

• Lastly, the paper would benefit from increased diversity and quantity in the quotes used, as not all ideas discussed in the text are supported by the evidence presented, either in the main text or the supplementary material, such as experiences with the disability identity card from ZAPD or the role of traditional healers. Furthermore, almost half of the quotes come from caregivers.

**We have added in additional quotes from clinicians to even the balance of quotes (e.g. line 645). We have aimed to provide quotes where they are salient and coherent.**
