# Supplementary material for: Access to healthcare for people with disabilities in Zambia: a qualitative study
Source: PLOS Glob Public Health. 2025 Mar 31;5(3):e0004145. doi: 10.1371/journal.pgph.0004145 (PMC11957284; doi:10.1371/journal.pgph.0004145)
Supplement: Attachment — Submitted filename: Response to reviewers_2025.docx [file pgph.0004145.s005.docx]

**Reviewer #1:**

I appreciate the authors' efforts in addressing the comments provided. While they chose not to implement certain suggestions, the manuscript is now well-structured and offers broader and more comprehensive conclusions. Congratulations on this valuable work and for amplifying the voices of people with disabilities—an essential contribution to public health research and advocacy.

**Thank you for your feedback. We very much appreciate your review and thank you for helping strengthen the final manuscript.**

**Reviewer #2:**

I appreciate the authors' efforts in incorporating the feedback into their work. This paper has many strengths and effectively highlights the voices and issues crucial to people with disabilities in Zambia. The importance of conducting this work cannot be overstated, as it sheds light on often-overlooked perspectives and challenges.

My main concern, however, is the lack of a second coder, which could affect the reliability of the findings. While the authors regularly discussed the coding process, which may be sufficient to ensure reliability, I wonder if you could find additional support in the literature for the use of a single coder.

Overall, this paper makes a valuable contribution to the field, advancing our understanding of disability-related issues in Zambia and offering important insights for future research and policy development.

**Thank you for your feedback on the single coder. We have made amendment in the methods (line 212 in tracked) and limitations (line 688 in tracked). Braun and Clarke in their new book do not recommend multiple coders to guarantee good analysis. They note that a single coder is good practice. We hope this is sufficient.**

**Than you for your overall review and for your time to help us improve the manuscript. We very much appreciate it.**
